# Allometry of Sexual Size Dimorphism in Domestic Dog

**DOI:** 10.1371/journal.pone.0046125

**Published:** 2012-09-25

**Authors:** Daniel Frynta, Jana Baudyšová, Petra Hradcová, Kateřina Faltusová, Lukáš Kratochvíl

**Affiliations:** 1 Department of Zoology, Faculty of Science, Charles University in Prague, Prague, Czech Republic; 2 Department of Ecology, Faculty of Science, Charles University in Prague, Prague, Czech Republic; Ecole Normale Supérieure de Lyon, France

## Abstract

**Background:**

The tendency for male-larger sexual size dimorphism (SSD) to scale with body size – a pattern termed Rensch's rule – has been empirically supported in many animal lineages. Nevertheless, its theoretical elucidation is a subject of debate. Here, we exploited the extreme morphological variability of domestic dog (*Canis familiaris*) to gain insights into evolutionary causes of this rule.

**Methodology/Principal Findings:**

We studied SSD and its allometry among 74 breeds ranging in height from less than 19 cm in Chihuahua to about 84 cm in Irish wolfhound. In total, the dataset included 6,221 individuals. We demonstrate that most dog breeds are male-larger, and SSD in large breeds is comparable to SSD of their wolf ancestor. Among breeds, SSD becomes smaller with decreasing body size. The smallest breeds are nearly monomorphic.

**Conclusions/Significance:**

SSD among dog breeds follows the pattern consistent with Rensch's rule. The variability of body size and corresponding changes in SSD among breeds of a domestic animal shaped by artificial selection can help to better understand processes leading to emergence of Rensch's rule.

## Introduction

Animal species that have undergone domestication processes usually exhibit rapid phenotypic change [1]–[4]. The extraordinary ability of domestic species to radiate into numerous morphologically and behaviorally distinct breeds within a few generations is explained by episodes of strong artificial selection [1], [5]. Domestic dog (*Canis familiaris*) is the most morphologically variable mammalian species [6]. Although dog is a product of multiple domestication events [7], its ancestors belonged to a single, morphologically rather uniform species, the wolf (*Canis lupus*). Differences in size are especially apparent among dog breeds, ranging from about two kilograms in the miniature Chihuahua to about 100 kilograms in the giant mastiff. This range encompasses the range of body mass reported for all other canids: from 1.3 kilograms in the fennec fox (*Fennecus zerda*) to 36.3 kilograms in the wolf [8]. Size differences, including those concerning differences between males and females, i.e., sexual size dimorphism (SSD), have many direct and indirect consequences [9], [10]. Exceptional magnitude of size variation and emerging knowledge on proximate mechanisms of body size changes [11]–[17] predetermines domestic dog to be a proper model for the assessment of allometric relationships.

In this study, we focus on allometry in SSD. Sexes differ in body size in many animal lineages [18], [19], but the relationship between female and male body size increase is usually not isometric. Males in larger species within an animal lineage tend to be larger relative to females than are males in smaller species. This empirically documented trend is currently referred to as Rensch's rule ([20], cf. [21], [22]). In recent years, this rule has attracted considerable research effort, and conforming patterns have been reported by interspecific comparisons in various animal taxa [20], [23]–[29], especially [30] or exclusively [31], [32] in taxa exhibiting male-larger SSD. The pattern consistent with Rensch's rule also has been demonstrated at an intraspecific level [33]–[36]. Several hypotheses have been formulated to explain Rensch's rule. Most popular has probably been the sexual selection hypothesis, being considered a general explanation for SSD allometry [27], [36], [37]. The sexual selection hypothesis suggests that Rensch's rule is driven by a correlated evolutionary change in female body size to directional sexual selection on increased body size in males.

Modern breeds of the domestic dog present a unique opportunity to test this hypothesis in the unusual situation where we know the selective agent responsible for size variation (artificial selection). The sexual selection hypothesis thus predicts that the pattern of SSD among dog breeds should not follow Rensch's rule. The aim of this paper is to examine the allometry of SSD and to test Rensch's rule among breeds of the domestic dog.

## Results

The Lovich-Gibbons ratios computed from the FCI standards [38] range from 1.00 up to 1.17 (mean  = 1.05), showing male-larger SSD in most breeds. SSD was positively correlated with female size irrespective of the trait used for its calculation (Spearman correlation coefficients *r_s_* = 0.29, *p*<.0001, *N* = 311 for shoulder height, and *r_s_* = 0.46, *p*<.0001, *N* = 159 for body mass).

In some breeds, FCI standards do not provide different values for stud dogs and bitches, and these theoretically monomorphic breeds tend to be smaller. It cannot be excluded that the absence of different size standards for males and females reflect just oversimplification during the process of standard formation. Therefore, we excluded these theoretically monomorphic breeds and computed the correlation coefficients again. The relationships did not remain significant after exclusion of the breeds with monomorphic standards (*r_s_* = −0.05, *p*<.48, *N* = 231 for shoulder height, and *r_s_* = 0.16, *p* = .14, *N* = 85 for body mass).

Next, we compared shoulder height of males and females based on our original data (Table S1). Mean shoulder height in males was larger than in females in 73 out of 74 breeds. ANOVA revealed that the variability in shoulder height can be explained by breed (*F*_73,6073_ = 5,449, *p*<.0001), sex (*F*_1,6073_ = 1,867, *p*<.0001), and their interaction (*F*_73,6073_ = 4, *p*<.0001). The Lovich-Gibbons ratios for shoulder height (mean  = 1.067, median  = 1.071) ranged from 0.995 in caniche toy to 1.099 in Slovakian hound. Size differences between the sexes were statistically significant (t-tests, *p*<0.05) in 69 breeds. Twenty-four breeds monomorphic according to the FCI standards appeared significantly dimorphic according to the original data. The sexual differences in shoulder height were not statistically significant in five extremely small breeds (Chihuahua, Prague ratter, papillon, miniature spitz, caniche toy). Moreover, t-tests failed to prove significant SSD for body mass in three of them (Chihuahua, Prague ratter, papillon) where sample size was larger than 17 individuals of each sex.

**Figure 1:**
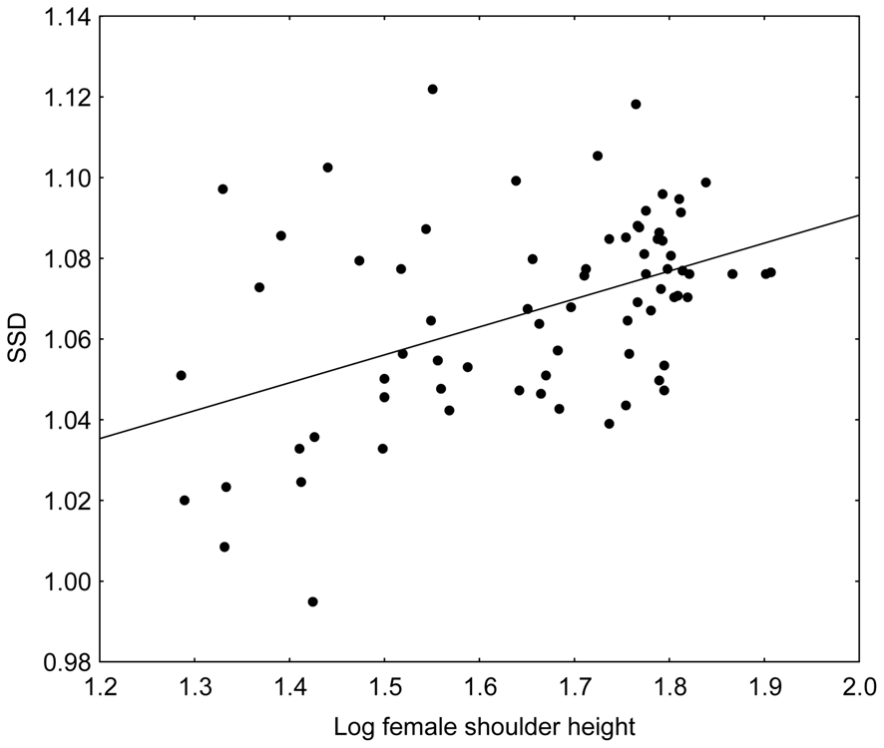
SSD increases with body size among dog breeds. Lovich-Gibbons ratios expressing SSD in shoulder height correlate with female shoulder height among 74 breeds of the domestic dog (*r* = 0.47; *p*<.0001).

**Figure 2:**
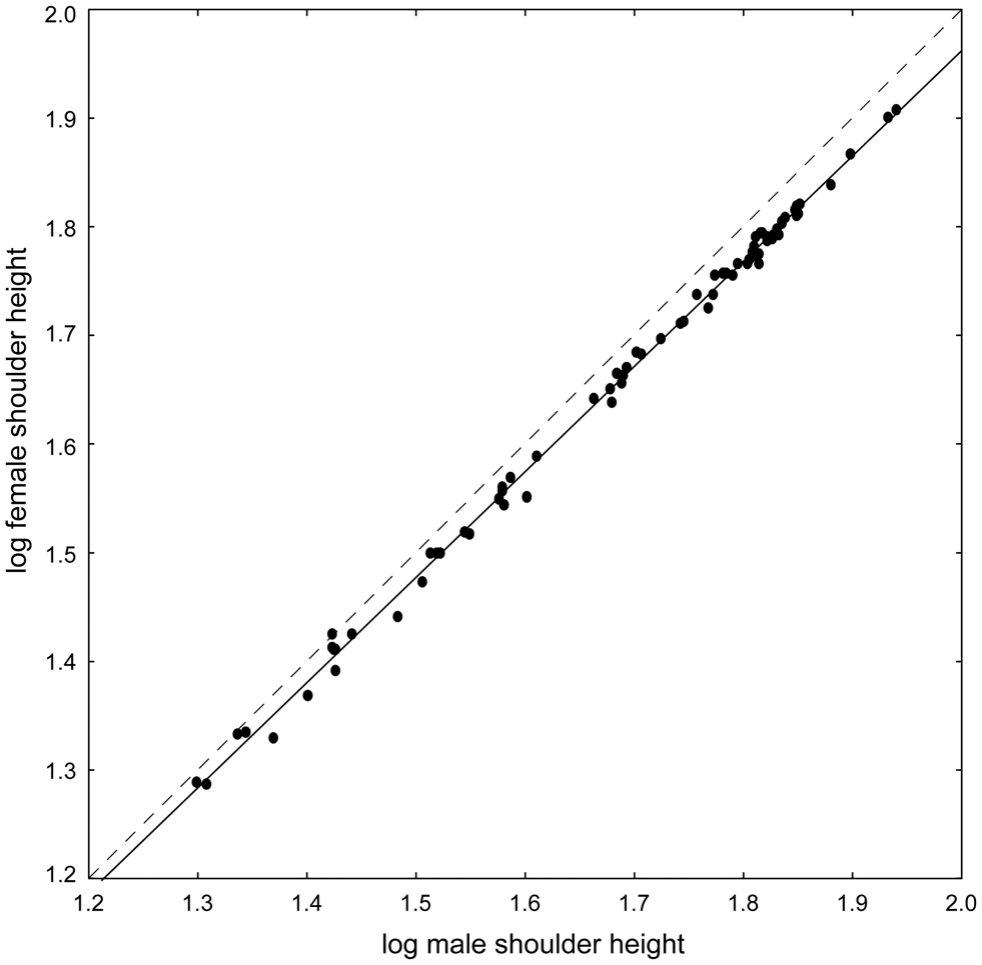
Relationship between mean male and female shoulder heights among 74 domestic dog breeds. Each point represents means computed from at least 10 males and 10 females. The data are naturally log-transformed. The slope of the fitted line is significantly less than 1, thus conforming to Rensch's rule. The broken line illustrates a 1:1 relationship.

Lovich-Gibbons ratios expressing SSD in shoulder height correlate with female shoulder height among 74 breeds of the domestic dog (*r* = 0.47; *p*<.0001; Figure 1) proving conformity with Rensch's rule among dog breeds. An alternative, but mathematically equivalent computation confirmed this trend. We found a clear linear relationship between log-transformed mean female shoulder height against log-transformed mean male shoulder height (Figure 2). The RMA slope of the line was 0.971 (95% confidence interval 0.956–0.982), which significantly deviates from the slope 1.0 expecting under isometry. The significant deviation from isometry remained unchanged when breeds represented by sample sizes smaller than 20, 30, and 50 individuals in at least one sex were excluded from the analyses; the corresponding values of the allometric slopes were 0.965, 0.956 and 0.961, respectively.

## Discussion

Our analysis confirms the pattern of SSD conforming to Rensch's rule among modern breeds of domestic dog. We were able to demonstrate significant allometry in SSD among breeds in our original dataset (Table S1). The results for data obtained from the FCI standards [38] confirmed Rensch's rule among breeds, as well, but many breeds treated in the standards as monomorphic proved to be in fact dimorphic based upon the real data. Our findings contradict the conclusions by Sutter *et al.* [39] that the breed standards rigorously adhered to morphological variation for domestic dog breeds. Sutter *et al.* [39] also concluded that the proportional size difference between males and females of small and large breeds in shoulder height is more or less the same, i.e., that breeds do not follow Rensch's rule (cf. to our Figures 1,2). The discrepancies between the studies can be explained by the different rules for including a particular breed into the respective dataset and different total number of studied breeds. Sutter *et al.* [39] included data for 53 breeds for which at least three males and three females were measured, while we analyzed data for 74 breeds with at least 10 males and 10 females measured.

Comparative analyses have long recognized the need to control for the non-independence of data that arises through patterns of shared common ancestry (phylogenetic non-independence). Ideally, the phylogenetic relationships among breeds should be taken into account to control for this effect in our test of Rensch's rule. Contemporary pedigree breeds have relatively short histories. Most of them were officially established in the 19th or even 20th century, and separation of their gene pools was thus completed only recently [40]. Consequently, mitochondrial [7], [41]–[45] and Y chromosome [46] haplotypes are distributed rather erratically among modern breeds. Microsatellite markers provide better resolution and allow genetic characterization of individual breeds [47], however, neither they can recover any reliable phylogenetic structure among the vast majority of modern breeds originating in Europe [40], [48]. Recently, an extensive SNP dataset allowed defining main genetic clusters of these breeds that fairly correspond to phenotypic/functional groups of breeds [49]. Nevertheless, these results are not in contradiction with earlier studies suggesting that the phylogenetic component represents only a small portion of genetic variation among modern dog breeds. Therefore, we assume that phylogenetic non-independence among dog breeds does not largely bias our conclusions.

The pattern consistent with Rensch's rule, i.e. larger evolutionary plasticity in body size in males than in females, among domestic dog breeds has emerged as a result of rapid radiation in size during domestication processes and subsequent formation of breeds. Interestingly, the domestication itself had no marked effect on the magnitude of SSD in domestic dog. Wolf, the direct ancestor of domestic dogs, is the largest species of the family Canidae and at the same time belongs to the most sexually dimorphic canids with male to female ratio in body mass about 1.28 [8]. This value fits well with male-to-female body mass ratios in dog breeds of comparable body size, such as hovawart (1.26), beauceron (1.25), giant schnauzer (1.22), or leonberger (1.21). Because the basal clades of canids comprise exclusively species of small, fox-like body size, the lineage leading to the genus *Canis* has been previously subject to substantial evolutionary increase in body size (cf. canid phylogenies in [50], [51]). On the other hand, according to the archaeozoological evidence, there was probably a great variability of size in dogs from the very beginning of the domestication process [52], [53]. Recent evolutionary trajectories of contemporary breeds probably involve changes in body size in both directions. Nevertheless, as the largest breeds of domestic dogs (e.g., great dane, Irish wolfhound) are only slightly larger than the largest subspecies of the wolf [54], the predominant direction in the evolution of body size in domestic dog was reduction rather than enlargement of the body size.

Thus, it seems that the pattern of SSD consistent with Rensch's rule among dog breeds evolved due to more pronounced size reduction in males in comparison to females. One can thus argue that the SSD allometry can be attributed to stronger selection on male than female size, and indeed, it seems that male dogs rather than bitches were the primary target of intentional artificial selection (cf. genetic evidence in [55]). Nevertheless, assuming that the size is primarily controlled by loci without sex-biased expression pattern, the genetic correlation between male and female body size should quickly eliminate the size difference caused by the higher selection pressure on a single sex [56]. Thus, it seems that Rensch's rule among domestic dog breeds can be rather attributed to more constrained female than male body size, especially in small breeds. There is some evidence that the presence of functional constraints represented mainly by size of an individual neonate limits further miniaturization of female size in small breeds. In large breeds such as hovawart, for example, the neonate mass relatively to female mass (1.45%) is about 75% smaller and the relative litter mass (12.2%) about 33% smaller than in Chihuahua (J. Baudyšová & D. Frynta, unpublished data). Moreover, breeders point out that bitches of the smallest breeds frequently suffer from complicated parturition.

The actual processes leading to pattern consistent with Rensch's rule among domestic dog breeds remain unclear. Nevertheless, the hypothesis on the functional constraint imposed by neonate size as a cause of SSD allometry consistent with Rensch's rule obtains support from the comparison between SSD allometry and reproductive allometry of domestic dog breeds and species of wild canids. The Lovich-Gibbons ratios computed from the data provided by Moehlmann and Hofer [8] do not correlate with female body mass (Spearman *r_s_* = 0.075, *p* = .688, *N* = 31 species,) among species of wild canids. Wild canids thus do not follow Rensch's rule, and, in accord with the hypothesis, small wild canids have much smaller neonate mass relatively to female body mass than do small dog breeds of the same size category. For example, neonate mass of 123 grams in Chihuahua (*N* = 62 newborns from 20 litters; J. Baudyšová, unpublished data) sharply contrast with 80 grams in *Vulpes corsac* [8]. The neonate mass relative to female body mass is about 1.5 times larger in Chihuahuas (5.9) than in corsac fox (3.9), while values obtained for hovawart (i.e., a breed resembling wolf in body mass) are well comparable to those in wolves [8], [57]. Differences in neonate mass scaling between wild canids and domestic dog breeds mirror different scaling in gestation length. Unlike in wild canids, which show a positive relationship between gestation length and body size [8], [57], gestation length is nearly the same across all domestic dog breeds irrespective of body mass [11], [58]. In view of that, it seems that during their evolution particular species of wild canids were able to adjust reproductive characteristics (gestation length, neonate size) according to female body size, while breeds of domestic dogs still possess values more similar to their relatively recent ancestor. These differences probably affect evolutionary plasticity in female body size and consequently also scaling of SSD. The difference in consistency with Rensch's rule between wild canids and domestic dogs are also concordant with the previously suggested scenario that Rensch's rule should be followed in lineages experiencing miniaturization in body size [24]. The common ancestor of wild canids was probably relatively small and the largest forms possess derived body size [8], [50], [51]. On the other hand, miniaturization was the predominant trend during formation of recent variability in body size among dog breeds from their wolf ancestor. Nevertheless, we should keep in mind that artificial selection and formation of breeds in domesticated animals is a different process involving for instance different genetic changes than speciation [59], and the comparison between body size radiations among dog breeds and in wild canids should be taken with caution.

In conclusion, we have demonstrated that the SSD pattern of domestic dogs follows Rensch's rule. Recently, SSD patterns consistent with Rensch's rule were demonstrated also in domestic cattle [60], goats and sheep [61], but not in domestic chicken breeds [37]. The evidence of Rensch's rule among breeds of a domestic animal shaped mainly by artificial selection supports a view that sexual selection cannot be considered as a general explanation for Rensch's rule. Rather, that evidence contributes to a more pluralistic view as to its formation. A pattern consistent with Rensch's rule seems to emerge in some clades exhibiting considerable evolutionary changes in body size, no matter whether they were driven by sexual, artificial or other type of selection.

## Materials and Methods

Male and female shoulder height and body mass were excerpted from the Féderation Cynologique Internationale (FCI) standards for particular breeds [38]. The standards provide data on 311 and 140 breeds for shoulder height and body mass, respectively. When the standards provide ranges instead of means or idealized/typical values, we used midpoints of minimum and maximum values.

Because the standards can be imprecise or biased, we also collected data on shoulder height in 6,221 dogs (2,714 males and 3,507 females) from 74 pedigree breeds. Each breed was represented by at least 10 individuals of each sex. All studied animals were included in studbooks of the Czech and Moravian Cynological Unit. They were measured in adulthood (i.e., at an age exceeding one year). The measurements were taken by the authors and/or authorities of particular breeding clubs (exhibition judges or breed advisors). The shoulder height was selected because (1) it is easily measurable and with high repeatability, (2) it does not depend on body condition, and (3) this measurement is the most correlated to the first principal component computed from a series of external measurements separately in three minutely studied breeds (briard, giant schnauzer; P. Hradcová, unpublished data; hovawart, J. Baudyšová & D. Frynta, unpublished manuscript). In morphometric studies, the first principal component often represents generalized body size. When available, data on body mass were recorded as well. Collection of the measurements was performed in accordance with Czech law implementing all corresponding European Union regulations and were approved by the institutional animal care and use committee (the Czech Ministry of Education, Youth and Sports No. 21384/2011- 30).

The SSD was expressed as the Lovich-Gibbons ratio [62] computed as M/F in male-larger breeds and 2-F/M in female-larger breeds. This ratio assures both linearity and proportional symmetry of SSD index (for details, see [63]). Significance of SSD was tested by t-tests and analysis of variance (ANOVA) with sex and breed as factors. Relationships between SSD and body size were tested by nonparametric or parametric correlations between the Lovich-Gibbons ratios and means of naturally log-transformed expressions of female size. Nonparametric tests were applied in the case of FCI standards, where distribution of SSD index deviated from normality. Means of naturally log-transformed data were further used in the estimation of interbreed allometries of female body size on male body size following [30]. Briefly, we estimated the slope of a regression of log-transformed mean female shoulder height against log-transformed mean male shoulder height. The slope 1.0 is expected under isometric increase of male and female size, while slope < 1.0 represents an increase in male-biased SSD with body size and thus the pattern correspondent with Rensch's rule. For the regression slope estimations, we employed the reduced major axis regression (RMA) model, which accounts for error in both dependent and independent variables [64]. Deviations from isometric relationship were considered significant when the expected isometric slope (1.0) fell outside the 95% confidence interval of the estimated slope. The calculations were performed using STATISTICA, version 6.0 [65].

## Supporting Information

Table S1 Descriptive statistics for male and female size in dog breeds.
(XLS)
